# The Absence of Abdominal Pigmentation in Livestock Associated *Culicoides* following Artificial Blood Feeding and the Epidemiological Implication for Arbovirus Surveillance

**DOI:** 10.3390/pathogens10121571

**Published:** 2021-12-02

**Authors:** Maria Goffredo, Michela Quaglia, Matteo De Ascentis, Silvio Gerardo d’Alessio, Valentina Federici, Annamaria Conte, Gert Johannes Venter

**Affiliations:** 1 Istituto Zooprofilattico Sperimentale dell’Abruzzo e del Molise G. Caporale, 64100 Teramo, Italy; m.goffredo@izs.it (M.G.); m.deascentis@izs.it (M.D.A.); s.dalessio@izs.it (S.G.d.); federicivale.vf@gmail.com (V.F.); a.conte@izs.it (A.C.); 2 Epidemiology, Vectors and Parasites, Agricultural Research Council-Onderstepoort Veterinary Research, Pretoria 0002, South Africa; venterjgert@gmail.com; 3 Department of Veterinary Tropical Diseases, University of Pretoria, Pretoria 0002, South Africa

**Keywords:** *Culicoides*, orbivirus, bluetongue, artificial blood feeding, abdominal pigmentation

## Abstract

*Culicoides* midges (Diptera: Ceratopogonidae), the vectors of economically important arboviruses such as bluetongue virus and African horse sickness virus, are of global importance. In the absence of transovarial transmission, the parity rate of a *Culicoides* population provides imperative information regarding the risk of virus dispersal. Abdominal pigmentation, which develops after blood feeding and ovipositioning, is used as an indicator of parity in *Culicoides.* During oral susceptibility trials over the last three decades, a persistent proportion of blood engorged females did not develop pigment after incubation. The present study, combining a number of feeding trials and different artificial feeding methods, reports on this phenomenon, as observed in various South African and Italian *Culicoides* species and populations. The absence of pigmentation in artificial blood-fed females was found in at least 23 *Culicoides* species, including important vectors such as *C. imicola*, *C. bolitinos*, *C. obsoletus*, and *C. scoticus*. Viruses were repeatedly detected in these unpigmented females after incubation. Blood meal size seems to play a role and this phenomenon could be present in the field and requires consideration, especially regarding the detection of virus in apparent “nulliparous” females and the identification of overwintering mechanisms and seasonally free vector zones.

## 1. Introduction

Unanticipated outbreaks of diseases caused by bluetongue virus (BTV; *Reoviridae*, *Orbivirus*) and Schmallenberg virus (SBV; *Peribunyaviridae*, *Orthobunyavirus*), and the subsequent overwintering of BTV in Europe [1,2] over the last decade, emphasized the necessity to clarify the epidemiology of these and other *Culicoides* (Diptera: Ceratopogonidae)-borne viruses [3,4,5,6,7,8,9,10,11,12]. This need was accentuated by a recent outbreak caused by African horse sickness virus (AHSV; *Reoviridae*, *Orbivirus*), in Thailand [13].

While both male and female *Culicoides* feed on nectar as a source of carbohydrates, females need a blood meal for the completion of their gonotrophic cycle and the production of eggs. Female *Culicoides* can therefore become infected while taking a blood meal from a viraemic animal. In susceptible females, the virus infects the midgut cells from where it is released into the haemocoel where it eventually infects the salivary glands [14]. Once the salivary glands are infected, the virus can be transmitted, via saliva, to a susceptible mammal host during subsequent blood feeding events for the rest of the insect’s lifespan [14].

In the genus *Culicoides*, the presence of an apparent ovarian barrier prevents the infection of the ovaries and transovarial transmission with viruses such as bluetongue and Akabane viruses [14,15,16,17]. Nulliparous females are thus not expected to be infected with viruses on emergence. It is therefore evident that only a subset of the female population, i.e., susceptible females that had fed on a viraemic host, will potentially be infected with viruses at any time. Although the number of infected females can be low, even in outbreak situations [18,19], this can supply valuable information regarding identifying potential vector species and the risk for the onwards spread of the virus. Taking into account that *Culicoides*, and especially *Culicoides imicola* Kieffer, can become very abundant during outbreak situations [20], coupled with a low expected infection rate, it will save time and resources if only parous females are tested for the presence of viruses. It is generally accepted that high proportions of parous females relate to a higher risk of virus transmission [21,22]. This phenomenon is considered within the European Regulation for the control, monitoring, surveillance, and restrictions on the movements of susceptible animals in relation to bluetongue. According to this regulation, a vector-free period is defined as the total absence of *C. imicola*, and less than five parous females of any other observed *Culicoides* vector species collected in light traps [23]. Monitoring of the parous rates in field populations contributes to an understanding of adult survival rates [24] and the voltinism of vector species [25,26].

From the above, it is clear that the reliable classification of parous *Culicoides* females is of the utmost importance. In 1969, Dyce, working in Australia, showed that *Culicoides* females deposit a burgundy pigment in the walls of the abdomen after digesting a blood meal and after the production of a batch of eggs. This pigment was absent in females that did not feed on blood, completed a gonotrophic cycle and produced eggs, as well as in males [27]. In the absence of transovarial transmission, unpigmented females can thus be considered as nulliparous and not expected to be infected with viruses. This pigment was found to be universal in the genus, the intensity of which increased with subsequent blood meals [28,29].

Although the chemical identity of the pigment is still unknown it was ascribed to ommochromes synthesized during egg development as a local waste product in the epithelial tissue. It was implied that the mechanism for waste disposal is likely related to poor circulation of haemolymph in the female abdomen, since it is distended and almost completely filled with the blood-engorged gut and developing eggs [29]. Determination and comparison of oral susceptibility, as a prerequisite for vector competence, of field populations of *Culicoides* infers the role these populations play in the transmission of viruses and the extent to which populations beyond the current range of an outbreak can contribute to the expansion thereof. As such, oral susceptibility studies were performed on field-collected *Culicoides* belonging to various European, Australian and South African populations using a variety of artificial feeding methods and viruses [30,31,32,33,34,35,36,37,38]. To determine to what extent viruses can persist and replicate in various livestock-associated *Culicoides* species, field-collected specimens were fed on a virus-infected blood meal in the laboratory and incubated for an appropriate extrinsic incubation time, before being tested for the persistence of the virus used for feeding. Oral susceptibility trials, conducted over the last two to three decades in South Africa and Italy, identify up to 13 South African [30,39,40] and at least three European *Culicoides* species [35,38], belonging to various sub genera, in which orbiviruses persist after extrinsic incubation, thereby indicating oral susceptibly and potential vector status. During these trials it was noticed that a persistent proportion of the blood engorged females did not develop any observable pigment after incubation.

**Figure 1:**
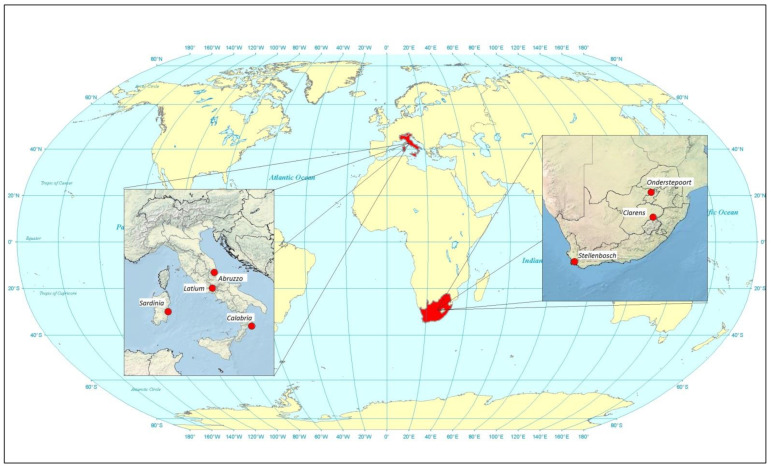
Collection sites in Italy and South Africa where *Culicoides* were collected with light traps for artificial blood feeding.

In the present study, we report on this phenomenon from a vast number of feeding trials, involving different artificial feeding methods and various *Culicoides* species and populations originating from different geographical areas (Figure 1) and timeframes. Based on their abundance near livestock, the most important vectors of arboviruses in Europe and Africa were considered to be *C. imicola*, *Culicoides bolitinos* Meiswinkel, *Culicoides obsoletus* (Meigen), and *Culicoides scoticus* Downes and Kettle, all belonging to the subgenus *Avaritia* Fox. The results related to the various species/populations are discussed in view of their implications for the entomological activities within arbovirus surveillance, as conducted in many countries.

## 2. Results

Age-grading results as obtained in oral susceptibly studies using different artificial feeding methods, were analyzed and compared. The presence or absence of abdominal pigmentation was determined in 36,495 females, 8 to 11 days after they had fed on a virus-infected blood meal (Table 1). The majority of these females, 30,924, originated from three sites in South Africa.

A total of 34 *Culicoides* species, 8 from Italy and 27 from South Africa, were considered. *Culicoides imicola*, the only species to be present in both areas, was the most abundant species in South Africa (21,405; 69.2%) and Italy (2263; 40.6%). The majority of the females, 30,775, were fed through a membrane system (M). The remainder, 5720, were fed on blood-soaked cotton wool pledgets (C). In the Italian studies, all the species, except for *C. imicola* (Sardinia), which was also fed through a membrane system, were subjected to cotton pledget feeding. A significant proportion was unpigmented upon completion of the incubation period. In Italy, the proportion of unpigmented females ranged from 8.6–55.4% for membrane and cotton-pledgets-fed females. In South Africa, it ranged from 10–41.5% (Table 1). Overall, the proportion of unpigmented (NP) females after incubation was significantly higher with C feeding (52.9%; 95% confidence interval (C.I.) 51.6–54.2%) compared to M feeding (9.9%; 95% C.I. 9.6–10.3%). The same significance was observed in the Italian and South African studies. In Italy, the proportion of NP with C feeding was 55.4% (95% C.I. 54.0–56.8%) compared to 8.6% with M feeding (95% C.I. 7.0–10.7%); in South African studies, the proportion of NP with C feeding was 41.5% (95% C.I. 38.5–44.6%), compared to 10% with M feeding (95% C.I. 9.7–10.3%) (Table 1).

**Figure 2:**
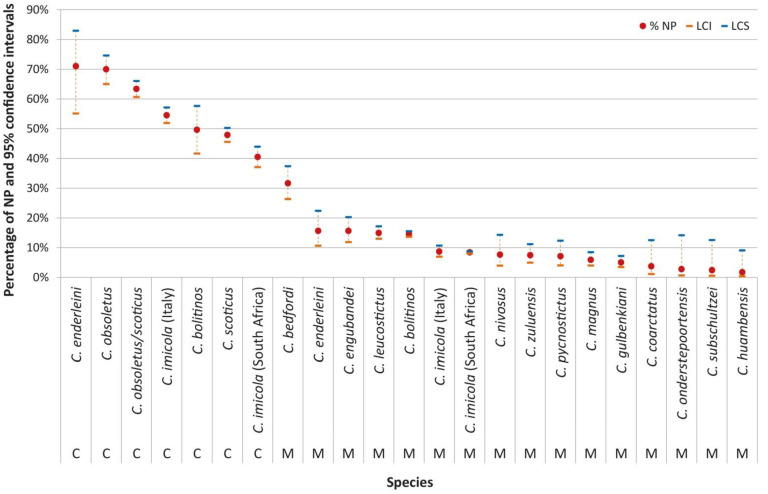
Percentage of females belonging to different *Culicoides* species, in South Africa and Italy with unpigmented abdomens 8 to 11 days after being fed either through cotton pledget (C) or membrane (M) (NP = not pigmented).

This pattern was confirmed within the species fed with both C and M methods, e.g., *C. imicola* (Italy and South Africa), *C. bolitinos*, and *Culicoides enderleini* Cornet and Brunhes (Figure 2). Within each cluster, corresponding to C and M methods, *C. imicola* (from both Italy and South Africa) showed significantly less unpigmented individuals than *C. bolitinos* with the M method, but the results overlapped with the C feeding (Figure 2).

Amongst the Italian species the proportions of NP females were significantly higher for *C. obsoletus* compared to those found in *C. scoticus* (Figure 2). Finally, the proportion of NP in Italian *C. imicola* fed with the C method was significantly lower than that for the *C. obsoletus*/*scoticus* taxon. This pattern was, however, only confirmed for *C. obsoletus* when the two single species were considered. Conversely, *C. imicola* NP were statistically higher than those of *C. scoticus* (Figure 2).

**Figure 3:**
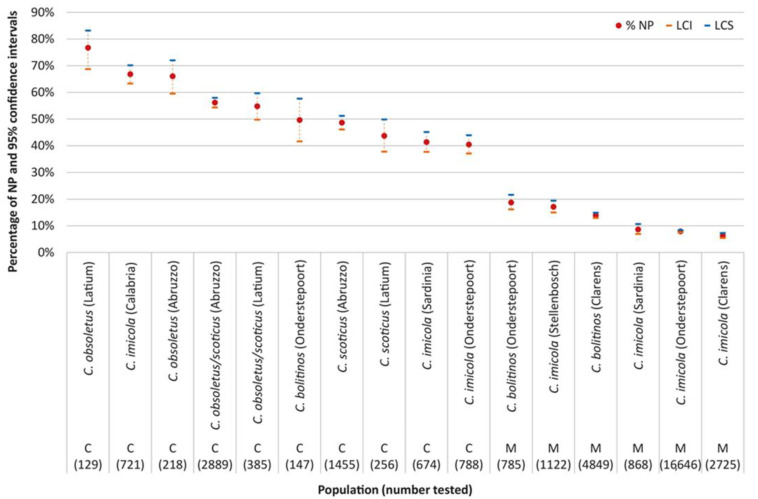
Percentage of females belonging to different *Culicoides* populations, in South Africa and Italy, with unpigmented abdomens 8 to 11 days after being fed either through cotton pledget (C) or membrane (M) (NP = not pigmented).

The proportional representation of NP was calculated for each vector population and interpopulation differences statistically evaluated (Figure 3). Overall, *C. imicola* from Italy and South Africa showed significant differences only with the C method, whereas they overlap with the M method (Figure 2). Within all the populations fed with both C and M methods (*C. imicola* Sardinia, *C. imicola* Onderstepoort and *C. bolitinos* Onderstepoort) a higher proportion of NP with the C method was confirmed.

Among the three populations of *C. imicola* fed with cotton pledgets, two from Italy and one from South Africa, the Calabria population showed a higher proportion of NP than Sardinia population, which in turn presented the same pattern of *C. imicola* from Onderstepoort. Within the four *C. imicola* populations fed on membranes, three from South Africa and one from Italy, the number of NP in the Stellenbosch population was significantly highest, whereas the confidence interval of Sardinia populations overlapped with Onderstepoort and Clarens. Regarding the two populations of *C. bolitinos*, fed on membrane, the population at Onderstepoort showed a significantly higher proportion of NP than the Clarens population. Between the two Italian populations of Latium and Abruzzo, no significant difference was detected when the taxon *C. obsoletus*/*scoticus* was considered, nor when *C. obsoletus* and *C. scoticus* were compared at the species level. Conversely, the significantly higher proportion of NP for *C. obsoletus* than *C. scoticus*, as above described as an interspecific pattern, is strongly confirmed within both the Abruzzo and Latium populations (Figure 3).

### 2.1. Comparison between Laboratory Feeding Results and Field Data

**Figure 4:**
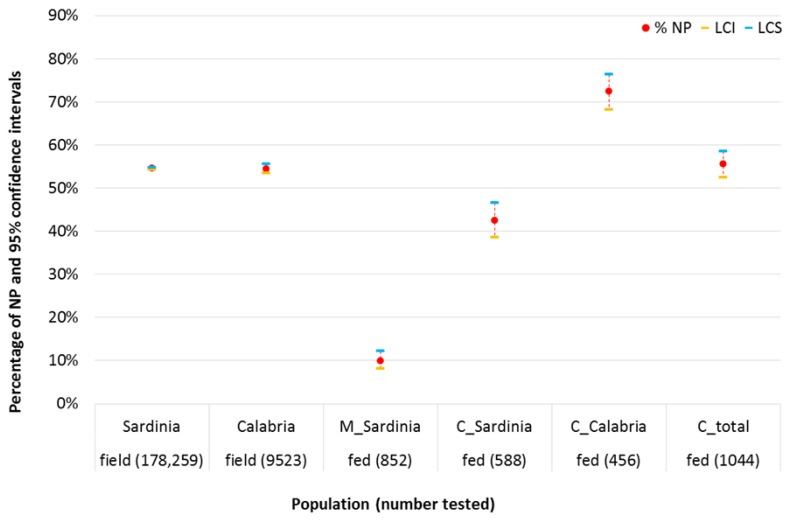
Abdominal pigmentation of two Italian *C. imicola* field populations, compared to that of live midges collected simultaneously and fed through cotton pledget (C) or membrane (M) 8 to 11 days after the blood meal (NP = not pigmented).

The age-grading results of the *C. imicola* populations as determined by five collections, each at Calabria and Sardinia, and the number of NP resulting from 21 feeding trials conducted simultaneously on live midges, are depicted in Figure 4. The proportion of unpigmented “nulliparous” females in two field populations of *C. imicola* did not differ significantly (Sardinia 95% C.I. 54.3–54.8%; Calabria 95% C.I. 53.5–55.5%). When the NP percentage after incubation was analyzed, a significant difference between the C and M feeding methods was observed: NP were significantly higher when the C method was used (M 95% C.I. 8.1–12.2%; C 95% C.I. 52.5–58.5%). This pattern was confirmed in the comparison of the two feeding methods within the Sardinia population. The percentage of NP in females fed with C method was similar, or even higher, than that obtained in the field in both populations. Conversely, with the M method, the number of NP clearly decreases compared to the field situation (Figure 4).

### 2.2. Repeated Feeding

A blood meal offered to 2168 field-collected NP *C. obsoletus*/*scoticus* from the Abruzzo population, through cotton wool pledgets, resulted in 767 fully engorged females. Of 520 surviving females, only 52 exhibited the typical abdomen pigmentation of “parous” females. Among the 468 females that remained NP after the first blood meal, 38 engorged for a second time, with 29 surviving 10 days. All, however, remained unpigmented. When a third blood meal was offered to them, only five of the eight females that engorged for a third time survived the 10 days of incubation. They, however, remained unpigmented (Table 2).

### 2.3. Virus Detection

Orally infected midges, without any visible abdominal pigmentation after incubation, were repetitively found to harbor the virus consumed through the artificial blood meal (Table 3). In particular, *C. bolitinos*, *C. imicola*, *C. scoticus* and *C. obsoletus* were positive for both BTV and AHSV strains. Interestingly, for all these vectors, the virus detection also occurred when the C method was used.

## 3. Discussion

During oral susceptibility studies conducted over the last three decades in South Africa and Italy it was found, in an apparent contradiction to the findings of Dyce (1969) [27], that persistent proportions of blood-fed, incubated females did not display any pigmentation after blood feeding and incubation. This phenomenon was, irrespective of the feeding method or virus involved, commonly observed in *C. imicola*, the only species tested in both continents, and in at least 23 South African and Italian *Culicoides* species (Table 1). In *C. imicola*, *C. bolitinos*, *C. obsoletus*, and *C. scoticus*, the most abundant vector species in Europe and Africa, the proportion of unpigmented (NP) females after incubation ranged from 8.4% to as high as 70% (Table 1). More significantly, the virus consumed through the artificial blood meal was repeatedly detected in these unpigmented females after incubation (Table 3). Of 34 species encountered, 11 did not show this phenomenon. Except for *C. milnei*, of which 29 individuals were tested, less than 10 specimens of the other 10 species were tested.

The European species included represent the pivotal vectors of *Culicoides*-borne diseases in Europe, such as BT and Schmallenberg, i.e., *C. imicola*, *C. obsoletus* and *C. scoticus*. *Culicoides obsoletus* and *C. scoticus*, two cryptic species belonging to the Obsoletus complex or “*C. obsoletus* group”, are considered the main vectors of orbiviruses in Northern and Central Europe, overlapping with *C. imicola* in Southern Europe [38,41,42].

The presence of unpigmented (NP) females, after an artificial blood meal in a relative great variety of South African species, indicates that this pattern may be common in the genus, as well as in European *Culicoides* species considered potential arbovirus vectors, such as *C. pulicaris*, *C. punctatus*, *C. newsteadi*, *C. dewulfi* and *C. chiopterus*. Although this hypothesis is partially supported for some of these species by the limited observations reported here, it must be confirmed by further studies.

Irrespective of the feeding method, statistical differences were observed among species. Although the reason for these differences is not clear it may be ascribed to differences in the biology and/or blood feeding response of the various species.

In addition to those observed between species, significant differences were found among various geographical populations of the same species. These differences indicate that the diverse environmental conditions, as experienced by the various populations before capture and feeding, may have contributed to the feeding success and subsequent development in the laboratory. For both the South African and Italian populations, the proportion of NP females was significantly higher for females that were fed via cotton pledgets than via membranes (Figure 2).

Female *Culicoides* need a blood meal to complete their gonotrophic cycle and it is accepted that one batch of eggs is matured after each blood meal is taken [43,44]. In the oral susceptibility studies, oviposition substrates were not provided to engorged females and no deposited eggs were observed. Eggs either did not develop or were reabsorbed in the absence of a suitable oviposition substrate. A comparison of feeding systems confirmed that the blood meal volume taken up during cotton pledget feeding (0.02 ± 0.01 μL), by females fed immediately after collection from the field, was significantly smaller than that taken up during membrane feeding (0.04 ± 0.01 μL) [45]. The impact of the smaller blood meal volume on egg development is not clear. The mean egg batch size of 6.8 eggs/*C. imicola* female after blood feeding using a membrane feeding system [46] was considerably smaller than that of 69 eggs/female reported by Nevill [47] after feeding on the shaven ear of rabbit, or that of 53–65 [48], 263 [44] or 69 eggs/female [49] in field-collected gravid *C. imicola* females. Overall, it seems that lower volumes of blood are taken up during artificial feeding compared to those on a host under natural conditions. The smaller blood meal size, especially during cotton pledget feeding, coupled with the absence of ovipositioning, may prevent the development of pigment in the blood-fed females. However, this observation will need to be confirmed.

Under natural conditions, it is usually accepted that blood feeding and egg production are interconnected. Oral susceptibility studies in the laboratory have, however, shown that this may not always be the case and that the eggs may be reabsorbed, or not formed, under unfavorable conditions. The relationship between blood feeding, egg production, and pigmentation needs to be clearly defined. The formation of pigment may be more related to egg production compared to blood feeding, as it also develops in some autogenous *Culicoides* species [50]. Further studies including the observation of abdomen pigmentation, coupled with the evaluation of midges to determine whether eggs were developed or whether they were later reabsorbed, would be beneficial.

The repetitive blood meals given to *C. obsoletus*/*scoticus* NP females in the present study demonstrated that at least 29 and 5 females remained unpigmented after a second and third blood meal, respectively (Table 2). It would be of interest to compare this observation with other vector species and with different feeding methods, in order to assess if females without pigmentation after the first blood meal would become pigmented following subsequent blood meals. In the American *C. variipennis*, it was shown that the intensity of the abdominal pigmentation increases in females that had two or more blood meals and completed two or more gonotrophic cycles. [28]. Despite the small number of midges tested in the present study, the findings support the hypothesis that more than one blood meal may be needed for the depositing of observable pigment in the abdomen of *Culicoides* females, and that unpigmented individuals collected in the field may already have had one or more blood meals, that in turn means a potential infection with arboviruses.

In vector competence studies the smaller blood meal size may relate directly to lower infection, or even a refractory status, in *Culicoides* reported for cotton pledget feeding [32,51]. The minimum virus titer in a blood meal needed to infect *Culicoides* species is not known. Bonneau [52], however, succeeded in infecting *Culicoides sonorensis* Wirth and Jones during feeding on a BTV-infected sheep with no detectable viraemia. This may imply that any titer of viraemia may be sufficient to establish an infection in at least a proportion of biting vectors. It is therefore possible that partially engorged females do not develop eggs and abdominal pigmentation, even though they become infected.

A Hemotek^®^ system was recently used to identify factors that may affect feeding success in the laboratory. It was shown that factors such as the source (host) and temperature of the blood meal, time of the day of feeding, the position of the blood reservoir in relation to the midges and exposure time to the blood may influence the volume of blood consumed by *C. imicola* [45]. In addition to the physiological condition of the females, other factors that potentially influence feeding success may include factors such as vibration, surface texture, skin, hair and feather thickness, carbon dioxide and other odor levels, visual stimuli, contact-chemical stimuli, and heat and moisture levels [53,54,55]. Although the potential extent of the influence of these factors of blood feeding under field conditions is unknown, it cannot be ignored.

The significantly higher proportion of NP females following C feeding is partly supported by the comparison of the parity rate in artificially fed females and field-collected females (Figure 4). When midges were fed with the C method, the NP% was similar to the field NP, and, at times, was even higher. Conversely, when midges were fed with the M method, the number of NP was distinctly lower compared to that in the field. Although this may be a reflection of the larger blood meal taken up during membrane feeding, it does not exclude the possibility that older pigmented field midges may be somewhat more experienced in blood feeding.

Irrespective of the reason, the present study indicates that virus can be detected from unpigmented females. Despite the absence of pigment, these females fed on a virus-infected blood meal in the laboratory. During the oral susceptibility trials only fully engorged females were selected, through visual inspection; nevertheless, the blood meal volume was possibly still smaller on cotton wool. Regardless, although this phenomenon is most likely a result of the smaller blood meals consumed under artificial conditions, it may still be present in the field. Considering the great number of factors that can influence the volume of blood consumed by a vector [45,53], it is not implausible that some individuals may indeed imbibe a below-average volume of blood, even under field conditions.

Depending on the trial and virus involved, virus detection was performed by virus isolation or real-time RT-PCR, testing insect bodies or heads individually (results not shown). Further studies are needed, specifically addressed to the evaluation of virus titers and ct values, found in unpigmented females, related to different combinations of vector species/virus strain. This could provide data to better understand the likely dissemination, and thus the ability of these unpigmented individuals to transmit the virus between hosts. However, it remains noteworthy that the virus detection after oral infection and an appropriate incubation period were not restricted to pigmented females, as shown in a number of trials involving diverse species and viruses (Table 3).

Although there are no reports of the isolation of any orbivirus from field-collected nulliparous *Culicoides* females, the detection of viral RNA of the Schmallenberg virus (*Orthobunyavirus*) from nulliparous European *Culicoides* species was reported [56], even from males [57]. In addition, it can be noted that potential venereal transmission with vesicular stomatitis virus (*Rhabdoviridae*) was recorded in *C. sonorensis* [58]. Although these findings, especially the presence of the virus in males, indicate potential transovarial transmission, it still need to be determined if these viruses will replicate to transmittable levels in these individuals. The extent to which the transovarial transmission is absent for orbiviruses also must be determined, as shown for the American *C. sonorensis* [17], and will be applicable to other viruses and European *Culicoides* species.

An apparent shortcoming of the use of pigmentation as an indicator of parity is that pigmentation may not always reflect the true parity status of a field population. The presence of abdominal pigmentation was reported in *Culicoides* collected in emergence traps [59,60]. Due to logistical reasons, virus detection attempts during outbreaks of viral diseases to determine field infection rates and to incriminate potential vector species, are usually restricted to pools of parous or “pigmented” females. To date, parous females were considered the best candidates since they were not freshly engorged and consumed and digested at least one potential virus-infected blood meal. The present results, however, suggest that unpigmented (NP) females can also indicate horizontal transmission, and active virus circulation in the area, since they are capable of becoming infected after feeding on a viraemic mammal host without depositing pigment in their abdomens. Considering that hundreds or thousands of midges can be collected in a single trapping event, sorting pigmented females for virus detection will remain advantageous. These pigmented females represent an older proportion of the females coupled to a greater possibility of exposure to infected hosts.

The achieved results suggest practical considerations, with regard to overwintering routes and seasonally free vector zones. Age-grading studies conducted on an Italian field population of *C. obsoletus*/*scoticus* indicate the presence of NP throughout the year, with the parity rate increasing from 10% in March (spring) to 56% in November (autumn) [41]. Midges belonging to this taxon, including nulliparous females, can be found feeding (endophagy) inside animal stables (endophily), a behavior encouraged by adverse weather conditions [61,62]. In addition, *C. obsoletus*/*scoticus* can survive for more than three months under laboratory conditions, and at least 10 days at fridge temperature (4 °C), without any blood meal [41]. The proportion of parous females is currently considered as the part of population possibly acting as vectors. Considering the present results, the portion of population capable of transmitting viruses should be extended to include a part of the nulliparous portion. This, combined with the endophilic behavior and resistance reported above, enhances the vector capacity of *C. obsoletus*/*scoticus* during adverse conditions and, most importantly, their capability to promote virus overwintering.

According to the European Regulation for the control, monitoring, surveillance and restrictions on movements of susceptible animals in relation to bluetongue, a vector-free period is defined as the total absence of *C. imicola* and less than five parous females of any other *Culicoides* vector species collected in light traps [23]. Although the present results, together with an apparent widespread susceptibly to orbiviruses in the genus *Culicoides* [63,64,65,66], suggest that this approach needs a re-evaluation, it should be emphasized that it is still unclear how widespread this phenomenon is in the field.

In conclusion, although differences were statistically recorded among species, populations or feeding methods, a portion of blood-fed midges without abdominal pigmentation was confirmed as a common pattern under laboratory conditions. Although the observed pattern seems to be linked to the feeding method, it is essential to better define the relationship between blood feeding, egg development and pigment production. Taking into account the great number of factors that can influence feeding success in the field, it cannot be excluded that this phenomenon will be present in the field. Further studies are needed, particularly to better investigate the effective capability of NP midges in transmitting the infection, through saliva, to susceptible hosts. Regardless, since virus detection repeatedly occurred in NP, after an artificial oral infection, the epidemiological role of the so called “nulliparous” in the field should be revised, particularly when it affects surveillance or control measures.

## 4. Materials and Methods

### 4.1. Specimen Collection and Artificial Feeding

The data originate from oral susceptibility trials conducted over the last two to three decades in South Africa and Italy, involving bluetongue, African horse sickness, equine encephalosis, and epizootic haemorrhagic disease viruses [30,32,35,38]. In these trials, alive midges were collected overnight with light traps in the vicinity of livestock [67]. To increase the variety of species evaluated and to incorporate diverse populations of the same species, collections were carried out in various geographical areas in South Africa, namely Onderstepoort, Clarens, and Stellenbosch (Figure 1). In Italy, collection sites were selected among the sites active within the entomological surveillance of BT, according to the abundance of *C. imicola* (Sardinia and Calabria) and *C. obsoletus*/*scoticus* (Abruzzo and Latium) (Figure 1).

After overnight collection, the midges were acclimatized in the laboratory for two to three days before being subjected to artificial blood feeding and attempted virus infection. The collected midges were maintained on a 5% to 10% sucrose solution on cotton pledgets, which were removed 24 h before blood feeding. Artificial feeding, using a one-day-old chicken skin or stretched Parafilm^®^ membrane on a defibrinated ovine or bovine blood/virus mixture, was conducted as described previously [30]. Due to a reluctance of European *Culicoides* species to feed through a membrane cotton pledgets feeding was evaluated and exploited as an alternative feeding method [32]. After a feeding time of 30–45 min the midges were immobilized at −20 °C and the blood engorged females sorted on an entomological chill-table. To minimize the time that the engorged females spent on the chill-table, and to increase survival, the blood engorged females were not identified to species level or age graded at this stage. Only fully engorged females, of which the abdomens were completely filled with fresh blood, were selected. These engorged females were maintained in a 5% to 10% sucrose solution and incubated for 8 to 11 days, at 23.5 °C to 25 °C and 40% to 80% R.H. (relative humidity). Surviving females were identified at species level and age graded [27] before being subjected to virus detection. South African *Culicoides* species were identified at species level using the wing picture atlas of Afrotropical *Culicoides* [68]. The identification of *C. obsoletus* and *C. scoticus* was performed by using an integrated morphological and molecular approach [41]. When the identification at species level was not performed, or was inconclusive, the specimens were labelled “*C. obsoletus*/*scoticus*”.

### 4.2. Comparison between Laboratory Feeding Results and Field Data

The original artificial feeding trials were based on field collections made over several days or even weeks. The natural seasonal fluctuation in the field parous rate [69] could have affected the proportions of parous females obtained after incubation. To overcome this potential shortcoming, the relationship between pigmented/unpigmented females, before and after the feeding of two Italian populations of *C. imicola*, were compared. During this comparison, midges were collected in parallel, either alive or in an appropriate collection medium [70].

At Sardinia and Calabria, three to six traps were operated per night at each farm to ensure sufficient numbers of *C. imicola* for artificial feedings. Over five nights, one trap at each site was used to collect insects in a collection medium [70], and all *C. imicola* females were age graded [27]. Living *Culicoides* were simultaneously collected at the same sites and subjected to membrane or cotton pledget feeding trials, as described. The blood-engorged females, obtained from each of the feeding trials, were age graded after the incubation, and the proportion of unpigmented (NP) nulliparous compared with that of the collections made in a collection medium. The percentage of unpigmented females from the field was calculated as the number of nulliparous individuals divided by the total number of midges collected, except engorged females.

### 4.3. Repeated Feeding

In the current study, field-collected *C. obsoletus*/*scoticus* females, from the Abruzzo population, in which no pigment was observed after an initial feeding, were fed for a second and third time and their pigmentation was recorded 10 days after each meal. Feeding was performed through cotton pledgets, and only the fully engorged midges were sorted and incubated at 25 °C and 40% to 80% R.H.

### 4.4. Virus Detection

Virus detection, as conducted during the original susceptibility trials, was performed by virus isolation on cell cultures or real-time RT-PCR depending on the trial. Most of the susceptibility results, obtained in the trials during which the pigmentation was recorded and shown in this current study, are already reported in the literature [35,38,39,40]. Here, we report only the findings of positive virus detection in pigmented females, orally infected under laboratory conditions.

### 4.5. Statistical Analyses

All comparisons were made using a Bayesian approach through the Beta distribution [71] which evaluates outcomes for percentages or proportions. The Beta distribution was calculated to express the percentage of pigmented midges (NP) in the compared groups and to assess the uncertainty of this estimate:

(1) $$Beta\left(\alpha_{1},\alpha_{2}\right)=\frac{x^{\alpha_{1}-1}{\left(1-x\right)}^{\alpha_{2}-1}}{\int_{0}^{1}t^{\alpha_{1}-1}{\left(1-t\right)}^{\alpha_{2}-1}dt}$$

where *α*_1_ = NP in tested samples + 1 (along the observation period); *α*_2_ = tested samples-NP in tested samples + 1. A 95% confidence interval was calculated for each parameter through the Beta distribution, and the differences were considered significant when the confidence intervals did not overlap. Only abundant species, representing >30 midges, were considered for statistical analyses.

## Figures and Tables

**Table 1:** Absence of abdominal pigmentation as determined in field-collected *Culicoides* species from Italy and South Africa, 8 to 11 days after being fed either through cotton wool pledgets (C) or membranes (M).

	C		M	
Species	No. of Tested	No. of NP * (%)	No. of Tested	No. of NP * (%)
Italy:				
*C. scoticus*	1711	820 (47.9)	-	-
*C. imicola*	1395	761 (54.6)	868	75 (8.6)
*C. obsoletus*/*scoticus*	1216	771 (63.4)	-	-
*C. obsoletus*	347	243 (70.0)	-	-
*C. circumscriptus*	6	0	-	-
*C. pulicaris*	16	4 (25.0)	-	-
*C. newsteadi*	5	4 (80.0)	-	-
*C. punctatus*	4	1 (25.0)	-	-
*C. montanus*	3	2 (66.7)	-	-
Total	4703	2606 (55.4)	868	75 (8.6)
South Africa:				
*C. imicola*	788	319 (40.5)	20,617	1735 (8.4)
*C. bolitinos*	147	73 (49.7)	5668	826 (14.6)
*C. leucostictus*	7	1 (14.3)	1136	170 (15.0)
*C. gulbenkiani*	-	-	537	27 (5.0)
*C. magnus*	-	-	427	25 (5.9)
*C. engubandei*	-	-	288	45 (15.6)
*C. zuluensis*	2	0	280	21 (7.5)
*C. bedfordi*	3	1 (33.3)	269	85 (31.6)
*C. pycnostictus*	12	1 (8.3)	154	11 (7.1)
*C. enderleini*	38	27 (71.1)	147	23 (15.6)
*C. nivosus*	18	0	105	8 (7.6)
*C. huambensis*	-	-	58	1 (1.7)
*C. coarctatus*	-	-	54	2 (3.7)
*C. subschultzei*	-	-	41	1 (2.4)
*C. onderstepoortensis*	-	-	36	1(2.8)
*C. milnei*	-	-	29	0
*C. dutoiti*	-	-	26	4 (15.4)
*C. nevilli*	-	-	14	2 (14.3)
*C. schultzei*	1	0	7	0
*C. neavei*	-	-	5	0
*C. expectator*	-	-	4	0
*C. angolensis*	-	-	1	0
*C. brucei*	-	-	1	0
*C. cornutus*	-	-	1	0
*C. nigripennis group*	-	-	1	0
*C. similis*	-	-	1	0
*C. tropicalis*	1	0	-	-
Total	1017	422 (41.5)	29,907	2987 (10.0)
Total Italy and SA	5720	3028 (52.9)	30,775	3062 (9.9)

* NP = not pigmented.

**Table 2:** Repeated blood feeding of *Culicoides obsoletus*/*scoticus*: not pigmented females fed two or three times (abdominal pigmentation was recorded 10 days post feeding on cotton wool pledgets).

Feeding	No. of NP * Females Tested	No. of Engorged Females (Feeding Rate %)	No. of Females Surviving 10 Days after Feeding	
			NP *	P **
1st	2168	767 (35.4)	468	52
2nd	468	38 (8.1)	29	0
3rd	29	8 (27.6)	5	0

* NP = not pigmented. ** P = pigmented.

**Table 3:** Virus detection from midges, fed through membrane (M) or cotton wool pledgets (C), without any abdomen pigmentation after 8 to 11 days incubation.

	African Horse Sickness Virus (AHSV)		Bluetongue Virus (BTV)		Epizootic Haemorrhagic Disease Virus (EHDV)	
Species	C	M	C	M	C	Total
*C. imicola*		12	49	47		108
*C. scoticus*	70		18		5	93
*C. bolitinos*		7	3	13		23
*C. obsoletus*	12		7		1	20
*C. leucostictus*		2				2
*C. magnus*		1				1
Total	82	22	77	60	6	247
